# Management of Low‐Energy Basicervical Proximal Femoral Fractures by Proximal Femoral Nail Anti‐Rotation

**DOI:** 10.1111/os.12579

**Published:** 2019-12-10

**Authors:** Qi Wang, Xiao‐hua Gu, Xi Li, Jian‐hong Wu, Yu‐feng Ju, Wei‐jie Huang, Qiu‐gen Wang

**Affiliations:** ^1^ Trauma Center, Shanghai General Hospital Shanghai Jiaotong University School of Medicine Shanghai China; ^2^ Trauma Center, Shanghai Seventh People's Hospital Shanghai China; ^3^ Trauma Center, Shanghai Punan Hospital Shanghai China

**Keywords:** Basicervical proximal femoral fractures, Elderly patients, PFNA‐II, Tip‐apex distance

## Abstract

**Objective:**

To evaluate clinical and radiological outcomes of proximal femoral nail anti‐rotation (PFNA‐II) devices and demonstrate the effectiveness of PFNA‐II for the treatment of basicervical fractures in elderly patients.

**Methods:**

A retrospective review of all patients treated with PFNA‐II for a proximal femoral fracture between January 2013 and February 2017 at three different institutions (Shanghai General Hospital, Shanghai Punan Hospital and Shanghai Seventh People's Hospital) was conducted. The X‐ray films were strictly reviewed by three trauma surgeons and a professional radiology doctor. Patients over 60 years of age who met the following criteria were included: (i) sustained low‐energy trauma; (ii) a two‐part fracture; (iii) fracture line located at the base of the femoral neck and that was medial to the intertrochanteric line and exited above the lesser trochanter but was more lateral than a classic transcervical fracture. Follow‐up time should be longer than 6 months. A total of 52 patients who met the inclusion criteria were selected. The average age at diagnosis was 75.1 years (range, 63–91 years); 13 patients were men and 39 were women. The same proximal femoral nail anti‐rotation devices and the same surgical procedures were applied to all patients. Postoperative radiographic union time and modified Harris hip scores were used as major indicators for evaluating the effectiveness of surgery.

**Results:**

The average follow‐up period was 22.5 months (18.5, 23.9, and 21.2 months, respectively) and radiographic unions were observed at an average of 19.6 weeks (range, 12–28 weeks). The patients were evaluated immediately after surgery, as well as 6 weeks, 3 months, 6 months, 1 year, and 2 years postoperatively. Of the 49 patients, 38 had good reduction qualities (75.5%), 9 acceptable (18.3%), and 3 poor (6.1%). Radiographic union was confirmed in all fractures at an average of 19.6 weeks (range, 12–28 weeks). The mean Harris hip score was 84.9 (range, 65–99): excellent in 9 patients (18.36%), good in 30 (61.22%), medium in 8 (16.32%), and poor in 2 (4.08%). Slight persistent pain occurred in 3 patients, but these patients could still walk with the help of a cane. Two patients had symptoms of excessive telescoping. Eight patients experienced postoperative medical complications, mainly pneumonia and urinary tract infection.

**Conclusion:**

Based on the clinical and radiological outcomes, the PFNA‐II devices provide strong rotational stability and excellent clinical prognosis, and are an appropriate treatment option for basicervical proximal femoral fracture in elderly patients.

## Introduction

With increasing age, the total bone mass of the human body decreases. Bone loss increases, and bone trabeculae become thinner, resulting in reduced bone strength^1^. Moreover, the general condition of older patients is often poor, due to accompanying cardiovascular diseases, lung diseases, and liver diseases. This can lead to a higher risk of fracture and postoperative complications.

In orthopaedic practice, femoral neck fracture (FNF) is a commonly encountered injury with high morbidity and mortality. It is evenly divided between femoral neck and intertrochanteric fractures, which account for approximately 3.6% of all fractures and are commonly encountered among the elderly and among young people who have sustained high‐energy trauma^2^. Basicervical proximal femoral fractures are a special type of femoral neck fracture, which are defined by Parker as a fracture in which the fracture line runs along the line of the anterior inferior attachment of the joint capsule and by Blair *et al* as a fracture in which the fracture line moves through the base of the femoral neck at its junction with the intertrochanteric region^3^, ^4^. In our article, in basicervical proximal femoral fractures, the fracture line is located at the base of the femoral neck and is medial to the intertrochanteric line and exits above the lesser trochanter but is more lateral than a classic transcervical fracture.

In the past 30 years, increasing attention has been paid to basicervical proximal femoral fractures. In the latest (2018) AO fracture classification, basicervical proximal femoral fractures were included for the first time, with a classification of 31B3^5^. This is a manifestation of the high interest in basicervical fractures and means that increasing attention is being paid to them. However, clinical defining and therapeutic methods of basicervical proximal femoral fractures are still in dispute. Due to the burdens of injury, long‐term immobilization and muscle atrophy may seriously damage the strength and power of fractured limbs, leading to serious complications such as deep vein thrombosis, thrombophlebitis, pulmonary embolism, urinary and pulmonary infections, and ulcers. Generally speaking, most patients prefer early surgical intervention^6^. Compared with other hip fractures, the basicervical fractures have greater biomechanical instability and higher incidence of implant‐related complications, which often leads to reoperation, increased mortality, increased length of hospital stay, increased difficulty of reoperation, higher failure rate, and higher cost. It is more difficult to treat patients with basicervical fractures.

The compression hip screw has traditionally been considered the “gold standard” for operative fixation of peritrochanteric fractures^7^. However, it is not an ideal choice for unstable fractures. Advances in cephalomedullary nailing (CMN) have led to better postoperative outcomes compared with the standard compression hip screw. The helical neck blade (PFNA) was determined as a better choice for fracture treatment, and had the ability to reduce the risk of bone loss and provide improved stability in the femoral head. An anti‐rotation screw can prevent rotation, which is beneficial for providing rotational control. The PFNA is characterized by exceptional healing ability and low incidence of major complications. However, the helical neck blade (PFNA) still has some shortcomings, such as being more prone to lateral cortex impingement^8^. Compared with PFNA, the proximal femoral nail anti‐rotation device (PFNA‐II) has the better mediolateral angle and a more flattened lateral surface, which consequently reduces the risk of fracture during insertion. It can provide stronger rotational stability and reduce the risk of secondary perforations (cut‐out). Compared with dynamic hip screw (DHS) devices, PFNA‐II is less invasive, requires less blood transfusion, and ensures mobilization of patients in a shorter time, and the operation duration is shorter. In the past ten years, the proximal femoral nail anti‐rotation (PFNA‐II) device has been more widely used, and surgeons have also chosen PFNA‐II to treat basicervical fractures. However, the literature regarding treatment of basal fractures with PFNA‐II is still very sparse and there are few data regarding treatment. In addition, the number of patients involved in existing studies is very small because of the scarcity of basicervical fractures^9^. Some of the conclusions of published studies are even conflicting. In 2016, an article by Watson and his team reported that the treatment of basicervical fractures with intramedullary nails was negated^10^. This surprised many clinicians and researchers and was the initial reason for our research. At the same time, debate about whether this method is reasonable is becoming more heated.

This article presents our experience in a series of 52 basicervical fractures treated with a proximal femoral nail antirotation device in three different institutions. One of the purposes of this article is to elaborate our understanding of basicervical proximal femoral fractures, including the definition and treatment of basicervical proximal femoral fractures. According to the clinical outcomes and incidence of implant‐related complications, this article also aims to evaluate clinical and radiological outcomes of proximal femoral nail anti‐rotation (PFNA‐II) devices and prove that PFNA‐II is effective in the treatment of basicervical proximal femoral fractures.

## Patients and Methods

### *Inclusion and Exclusion Criteria*

Approval from the institutional review board and patients' informed consent were obtained for this retrospective study.

We reviewed radiographs and medical records of all patients treated with PFNA‐II for a proximal femoral fracture between January 2013 and February 2017 at three different institutions: Shanghai Punan Hospital, Shanghai General Hospital, and Shanghai Seventh People's Hospital. The X‐ray films were reviewed by three trauma surgeons from the department of orthopaedics and a professional radiology doctor to identify basicervical proximal femoral fractures.

Patients in conformity with the following criteria were included: (i) age older than 60 years; (ii) sustained low‐energy trauma; (iii) a two‐part fracture; (iv) fracture line located at the base of the femoral neck that was medial to the intertrochanteric line and exited above the lesser trochanter but was more lateral than a classic transcervical fracture (Fig. 2); (v) treated with PFNA‐II; and (vi) follow‐up time exceeded 6 months.

Through reviewing the medical records, patients in conformity with the following criteria were excluded: (i) sustained high‐energy trauma; (ii) any suspicion of pathological fracture; (iii) the lesser trochanter was a separate fragment or the fracture line exited distal to the lesser trochanter or out the lateral cortex of the greater trochanter; (iv) treatment with other internal fixators; and (v) the follow‐up time was less than 6 months.

**Figure 1:**
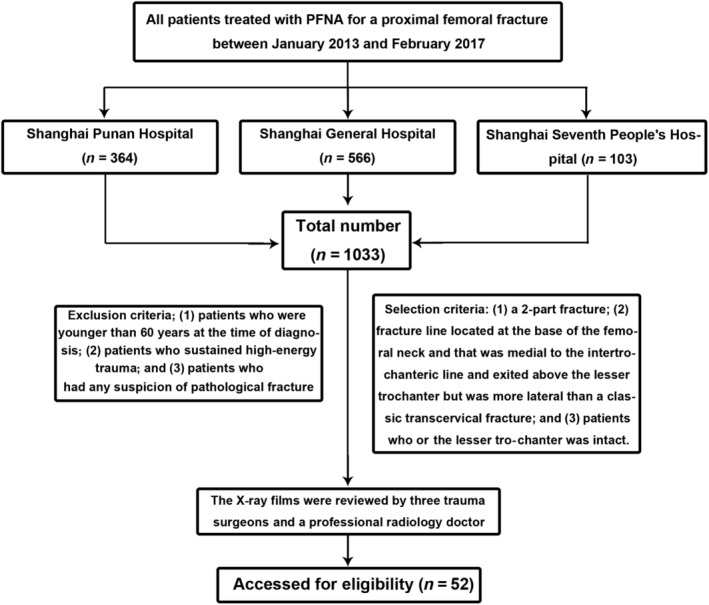
The flow diagram of subject investigated. PFNA, proximal femoral nail anti‐rotation.

Finally, 1652 proximal femoral fractures at three institutions during the specified period were reviewed. A total of 1033 patients were treated surgically with the same single helical blade CMN system (DePuy Synthes PFNA‐II Asian). The whole process is detailed in Fig. 1. Sitting was allowed from the first postoperative day, and weight‐bearing as tolerated began on the second or third day after the operation.

**Figure 2:**
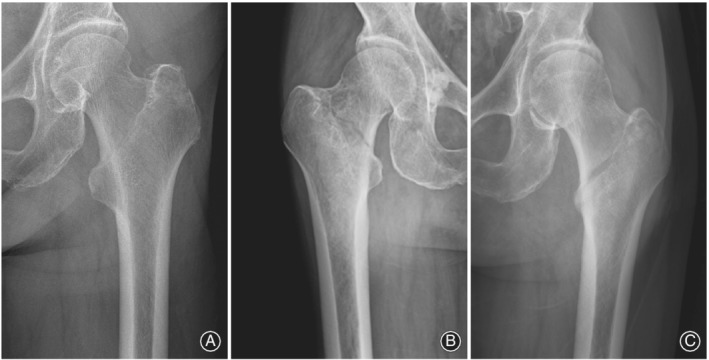
Radiograph of a typical basicervical intertrochanteric fracture: (A) Shanghai Punan Hospital, (B) Shanghai General Hospital, and (C) Shanghai Seventh People's Hospital.

### *Postoperative Evaluation Parameters*

#### *Reduction Quality*

**Figure 3:**
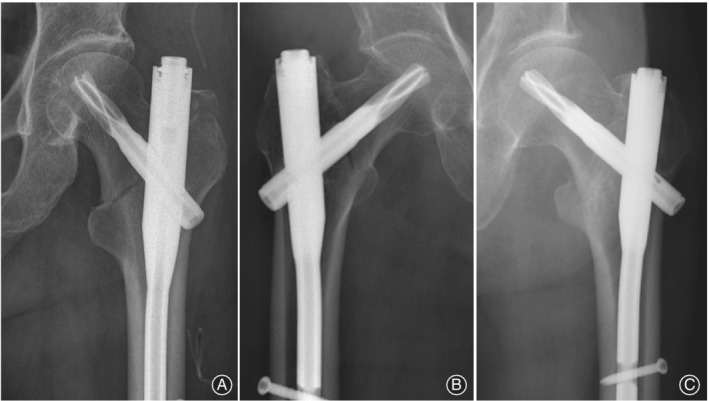
Postoperative radiograph of a typical basicervical intertrochanteric fracture: (A) Shanghai Punan Hospital, (B) Shanghai General Hospital, and (C) Shanghai Seventh People's Hospital.

Reduction quality was evaluated by intraoperative and postoperative fluoroscopic images, which were graded as good (<5 varus/valgus and/or anteversion/retroversion), acceptable (5‐10), or poor (>10) (Fig. 3)^11^.

#### *The Tip–Apex Distance*

The blade was directed to the center–center position and the tip–apex distance (TAD) of all helical blades was measured on the first postoperative X‐ray images to assess blade position. TAD usually refers to the sum of the distance in millimeters from the tip of the lag screw to the apex of the femoral head as measured on an anteroposterior radiograph and that distance on a lateral radiograph, after correction for magnification^12^.

#### *Postoperative Complications*

The patients were evaluated at 6 weeks, 3 months, 6 months, 1 year, and 2 years postoperatively with clinical and radiographic assessment of the progress of healing and assessment of complications. The major postoperative complications were identified as cut‐out/cut‐through, hardware‐related femoral fracture, nonunion of the fracture, movement of the position of the lag screw in the femoral head, and varus development as a result of collapse. The diagnoses of postoperative complications were mainly dependent on postoperative and follow‐up imaging.

#### *The Modified Harris Hip Score*

Upon clinical assessment, the modified Harris hip score was measured at the final follow up. The modified Harris hip score (mHHS) is a joint‐specific score, which covers domains of pain, function (limp, support, and distance walked), functional activities (stairs, squatting, sitting cross legged, and public transportation), deformity, and hip range of motion^13^. Evaluation of the modified Harris hip score was similar to Harris hip score: <70 (poor result), 70–79 (fair result), 80–89 (good result), and > 90 (excellent result).

## Results

### *Study Search*

After reviewing the records of 1652 patients with proximal femoral fractures between January 2013 and February 2017 at three different institutions, 1033 patients with PFNA‐II were screened out (364, 566, and 103 patients, respectively). After systematic evaluation, 52 patients met the criteria of basicervical fracture treated with a PFNA‐II device: 4 from Shanghai Punan Hospital (3.80%), 29 from Shanghai General Hospital (5.12%), and 19 from Shanghai Seventh People's Hospital (5.21%). Among them, 13 were men and 39 were women (mean age, 75.1 years; range, 63–91 years). All fractures were a consequence of a low‐energy mechanism of injury, such as a fall from a standing height. A total of 27 and 25 patients had right and left hip fractures, respectively (Table 1).

**Table 1:** Preoperative patient data

Institution	Male	Female	Average age (years)	Left fractures	Right fractures
Shanghai Punan Hospital	1	3	72.5 (range, 63–78)	2	2
Shanghai General Hospital	9	20	78.9 (range, 64–91)	13	16
Shanghai Seventh People's Hospital	3	16	69.9 (range, 64–85)	12	7
Total	13 (25%)	39 (75%)	75.1 (range, 63–91)	27	25

### *Follow‐up*

The evaluation intervals of all patients were similar (immediately after surgery, at 6 weeks, 3 months, 6 months, 1 year, and 2 years postoperatively). The mean follow‐up time was 22.5 months (18.5, 23.9, and 21.2 months, respectively). During follow‐up, 1 of the 52 patients died less than 6 weeks after injury, and 2 did not return for follow‐up after discharge from the hospital. The remaining 49 patients were included in the analysis.

### *General Results*

The average time for the operation was 63.4 min. There were no revision surgery cases. No intraoperative complications such as loss of reduction, displacement of the fragments during implant insertion, or intraoperative fracture occurred.

### *Radiographic Evaluation*

According to postoperative radiographs, the reduction qualities of 38 of the 49 patients were good (75.5%), 9 were acceptable (18.3%), and 3 were poor (6.1%) (Table 2). Nearly 86.5% of patients had an ideal implant position. The mean TAD of immediate postoperative radiographs was 21.8 mm (range, 18.2–24.7 mm).

**Table 2:** Quality of fracture reduction and postoperative radiographic evaluation

Institutions	Good	Acceptable	Poor	Ideal implant position (%)
Shanghai Punan Hospital	3	1	0	100
Shanghai General Hospital	20	5	2	82.6
Shanghai Seventh People's Hospital	14	3	1	89.4
Total (%)	75.5	18.3	6.1	86.5

Radiographic union, as evidenced by bony trabeculae crossing the fracture interspace, was confirmed in all fractures at an average of 19.6 weeks (range, 12–28 weeks), but excessive telescoping was found in 2 patients: 12.3 mm in a 78‐year‐old woman and 11.9 mm in an 82‐year‐old woman.

### *Functional Evaluation*

The modified Harris hip score was used to evaluate the function of the hip joint postoperatively. According to the final follow up, the mean Harris hip score was 84.9 (range, 65–99); the score was excellent in 9 patients (18.36%), good in 30 (61.22%), fair in 8 (16.32%), and poor in 2 (4.08%) (Table 3).

**Table 3:** Evaluation of PFNA‐II for the treatment of femoral basicervical fractures

Institutions	Average operation time (min)	Mean TAD (mm)	Time for radiographic union (months)	The modified Harris hip score				
				Scores	Excellent	Good	Fair	Poor
Shanghai Punan Hospital	73	20.3 (range, 19.6–22.2)	18.5 (range, 16–26)	91	0	2	2	0
Shanghai General Hospital	59	21.4 (range, 18.2–24.5)	18.9 (range, 12–24)	83.7	6	17	3	1
Shanghai Seventh People's Hospital	68	22.7 (range, 20.2–24.7)	20.9 (range, 16–28)	85.5	3	11	3	1
Total	63.4	21.8 (range, 18.2–24.7)	19.6 (range, 12–28)	84.9	9	30	8	2

TAD, tip–apex distance.

### *Complications*

Most fractures had healed with no loss of position by the 6‐month check‐up. No cut‐out or cut‐thorough of the helical blade was observed. Slight persistent pain occurred in 3 patients, possibly due to prominent implants, but these patients could still walk with the help of a cane, and there was no need for implant removal.

Eight patients experienced postoperative medical complications, mainly pneumonia and urinary tract infection. Significant improvement was achieved after symptomatic treatment.

## Discussion

In China, the aging population is leading to a significant increase in the incidence of transcervical fractures, intertrochanteric femoral fractures, and basicervical proximal femoral fractures. Due to their poor bone quality, it is more difficult to achieve and maintain a stable fixation in elderly patients. The best method to effectively treat basicervical proximal femoral fractures is becoming a hot topic of debate.

### *Current Status of Treatment*

As early as 2013, in the study of Hu *et al*.^12^, intramedullary nailing was reported for the treatment of basicervical fractures. Almost all of 32 patients achieved satisfactory results. However, in the study of Watson *et al*. (2016)^10^, the effectiveness of intramedullary nails on basicervical fractures was not supported. Among 11 patients, 6 patients had complications after the operation, and the failure rate was reported to be 54.5%. In addition, Waston *et al*. questioned the high success rate and satisfaction rate of Hu in his article. They noted that in Hu's article, the patients included some young adults, with basicervical fractures of 10 patients resulting from high‐energy injuries. Young people have higher bone density and faster recovery, which are reasons for better prognosis. Another important factor was that Watson thought Hu's definition of basicervical fractures was inaccurate. Watson *et al*. strictly defined the basicervical fractures as occurring at the base of the femoral neck and exiting above the lesser trochanter. To fully demonstrate the effectiveness of PFNA‐II in the treatment of basicervical fractures, we also adopted only basicervical fractures which conformed to this definition.

After Waston's article, there were increasing numbers of studies about the suitability of intramedullary nails for basicervical fractures. In 2017, Okano *et al*. conducted a retrospective review of 500 consecutive cases between January 2005 and February 2015. A total of 16 cases met the definition and only 2 cases had relatively minor mechanical complications^14^. Kim *et al*. (2019) compared the effects of different fixation devices on the treatment of basicervical fractures. They proved that CMN with blade type was more suitable for basicervical fractures^15^.

In the field of biomechanical studies, through finite element analysis, Kwak *et al*. compared three different intramedullary nails for fixation of unstable basicervical fractures. Compared with other fixation devices, PFNA‐II had the greatest failure load and structural stability. PFNA‐II was also more effective in minimizing the rotation instability of the proximal fragment in basicervical fractures^16^.

In this study, we used a new helical blade CMN system (PFNA‐II), which was different from the intramedullary devices with lag screw constructs in the study of Watson. The helical neck blade (PFNA, AO/ASIF), the primary innovation of the proximal femoral nail anti‐rotation design, is capable of reducing the risk of bone loss and providing improved stability in the femoral head as a result of compaction of cancellous bone around the blade during insertion^8^. The PFNA is characterized by exceptional healing ability and low incidence of major complications; nevertheless, it has been associated with lateral cortex impingement. It effects lateral cortex fractures and fracture displacement during insertion. The round profile of the nail creates pressure on the lateral wall and the head–neck fragment, leading to the damage of the lateral wall and the loss of reduction and varus of the head–neck fragment, which is a complication that decreases stability and increases the risk of cut‐out. The PFNA II was designed to solve those potential concerns. The mediolateral angle of PFNA II is reduced to 5°, allowing a slightly more lateral entry point through the tip of the greater trochanter. On top of that, a more flattened lateral surface that theoretically decreases the length of the region of impingement on the lateral cortex is offered by PFNA II, which consequently reduces the risk of fracture during insertion. It can act as an internal splint to bear a large axial load, and the helical blade can also enhance its bone purchase in the femoral neck–head and prevent rotation or compaction of the proximal fragment by locking the nail rotationally^9^. Biomechanical studies show that the helical blade has higher rotational stability compared with lag screws. In addition, due to a modified screw or blade design and an improvement in the sliding properties of the femoral neck components, PFNA‐II devices promise a stronger hold in osteoporotic bone with a lower rate of secondary perforations (cut‐out) by the implant in the head–neck fragment^17^. Compared with dynamic hip screw (DHS), PFNA‐II devices can also provide stable biological support and improve fracture healing. PFNA‐II devices require a relatively smaller exposure, less tissue handling, and less anatomical reduction, all of which could decrease morbidity, the probability of infection and significant blood loss, the possibility of varus collapse, and the inability of the implant to survive until fracture union. With these excellent properties, we believe PFNA‐II devices are an ideal choice for the treatment of basicervical fractures, providing stable anti‐rotation and anti‐sliding properties, which play a great role in preventing cut‐out and providing stable biological performance^18^.

### *Influencing Factors*

The TAD was first described by Baumgaertner *et al*.^11^, and they found that patients with a TAD >25 mm were more likely to have cut‐outs. Although the fixation device they used was SHS, TAD has been applied for almost all devices, including CMN. It is generally believed that when TAD is less than or equal to 25 mm, the spiral blade is close to the subchondral bone, where the bone is relatively denser. When TAD is greater than 25 mm, because of the cancellous bone there, the blade moves more frequently in the femoral head after loading and is prone to internal fixation failure, such as coxa adducta. Recently, however, there has been some doubt about the reliability of the TAD value. Watson questioned the rationale of the TAD value, because many patients still had complications of cut‐out even with an ideal TAD value in his study. For this problem, early in the study of Goffin *et al*.^19^, it was found that lag screw cut‐out occurs because the bone superior to the lag screw thread is too weak to sustain compressive strains exerted by the lag screw. The incidence of this condition is not only dependent on TAD but also closely related to the size of the femoral head and the placement of lag screws. In the study of Hsueh *et al*., it was also proven that even in the TAD range from 25 mm to 45 mm, only a few patients (17%) had cut‐outs^20^. However, in patients with below 25 mm, the incidence was also significantly reduced. The FNF at the base of the neck is often obscured by the tuberosity, which makes the fracture line invisible. This shows that the TAD value is not a key indicator of postoperative cut‐out complications, but a reasonable TAD value still helps us to get a better prognosis.

Early functional exercise is recommended in many studies, but due to too much shear stress at the fracture site, premature load commitment may affect the healing of the fracture and postoperative complications for patients with basicervical fractures. Kweon *et al*. suggested that in the course of rehabilitation training^21^, sitting should be allowed from the first postoperative day, and wheelchair usage and partial weight‐bearing was indicated between the 3rd and the 7th postoperative days, depending on the degree of reduction, systemic conditions, and pain^22^.

### *Limitations of the Study*

First, the small number of patients is an obvious deficiency in our study, as with all current studies on basicervical fractures. More date may make our conclusion more convincing. After establishing a precise definition of basicervical fractures, the number of patients was reduced, so it was difficult to conduct large sample analysis^22^. Second, this is a retrospective study with no control group. Although there are no comparative studies that demonstrate the difference between PFNA‐II and other devices, it was at least an indication that PFNA‐II was an effective method for basicervical fractures. Third, our study population included only East Asians.

New further research is in the pipeline. We will design more rigorous prospective clinical studies to compare the efficacy of PFNA‐II and other intramedullary devices for basicervical fractures to further investigate the appropriate treatment. In diagnosis, X‐ray alone is often not enough for accurate diagnose of basicervical fracture. We also consider CT examination of patients to further standardize the definition of basicervical fractures.

### *Conclusion*

This study examined the use of PFNA‐II for the treatment of basicervical fractures, suggesting that this type of implant may be an appropriate choice for this fracture type. We believe that PFNA‐II fixation is currently the optimal procedure for basicervical proximal femoral fractures.
